# Associations of plasma proteomics and age-related outcomes with brain age in a diverse cohort

**DOI:** 10.1007/s11357-024-01112-4

**Published:** 2024-03-04

**Authors:** Ramon Casanova, Keenan A. Walker, Jamie N. Justice, Andrea Anderson, Michael R. Duggan, Jenifer Cordon, Ryan T. Barnard, Lingyi Lu, Fang-Chi Hsu, Sanaz Sedaghat, Anna Prizment, Stephen B. Kritchevsky, Lynne E. Wagenknecht, Timothy M. Hughes

**Affiliations:** 1 Department of Biostatistics and Data Science, Wake Forest University School of Medicine, Medical Center Blvd., Winston-Salem, NC USA; 2 National Institute of Health, Baltimore, MD USA; 3 Department of Internal Medicine, Wake Forest University School of Medicine, Winston-Salem, NC USA; 4 School of Public Health, Oncology and Transplantation, University of Minnesota, Minneapolis, MN USA; 5 Division of Epidemiology and Community Health, School of Public Health, University of Minnesota, Minneapolis, MN USA; 6 Division of Public Health Sciences, Wake Forest University School of Medicine, Winston-Salem, NC USA

**Keywords:** Machine learning, Brain age, Alzheimer’s disease, Machine learning, Proteomics, Mortality

## Abstract

Machine learning models are increasingly being used to estimate “brain age” from neuroimaging data. The gap between chronological age and the estimated brain age gap (BAG) is potentially a measure of accelerated and resilient brain aging. Brain age calculated in this fashion has been shown to be associated with mortality, measures of physical function, health, and disease. Here, we estimate the BAG using a voxel-based elastic net regression approach, and then, we investigate its associations with mortality, cognitive status, and measures of health and disease in participants from Atherosclerosis Risk in Communities (ARIC) study who had a brain MRI at visit 5 of the study. Finally, we used the SOMAscan assay containing 4877 proteins to examine the proteomic associations with the MRI-defined BAG. Among *N* = 1849 participants (age, 76.4 (SD 5.6)), we found that increased values of BAG were strongly associated with increased mortality and increased severity of the cognitive status. Strong associations with mortality persisted when the analyses were performed in cognitively normal participants. In addition, it was strongly associated with BMI, diabetes, measures of physical function, hypertension, prevalent heart disease, and stroke. Finally, we found 33 proteins associated with BAG after a correction for multiple comparisons. The top proteins with positive associations to brain age were growth/differentiation factor 15 (GDF-15), Sushi, von Willebrand factor type A, EGF, and pentraxin domain-containing protein 1 (SEVP 1), matrilysin (MMP7), ADAMTS-like protein 2 (ADAMTS), and heat shock 70 kDa protein 1B (HSPA1B) while EGF-receptor (EGFR), mast/stem-cell-growth-factor-receptor (KIT), coagulation-factor-VII, and cGMP-dependent-protein-kinase-1 (PRKG1) were negatively associated to brain age. Several of these proteins were previously associated with dementia in ARIC. These results suggest that circulating proteins implicated in biological aging, cellular senescence, angiogenesis, and coagulation are associated with a neuroimaging measure of brain aging.

**Supplementary Information:**

The online version contains supplementary material available at 10.1007/s11357-024-01112-4.

## Introduction

Estimation of measures of “brain age” from neuroimaging data [1–4] using machine learning models is becoming a popular approach to derive measures of brain age [2, 5]. While multimodal data can be used to estimate brain age [4], it is often derived from structural MRI because of its availability. The difference between estimated brain age and chronological age called brain age gap (BAG) is potentially a measure of brain health [2, 5, 6], which may have clinical utility, as it has been associated with cognitive impairment and progression to Alzheimer’s disease (AD) [7], mortality, and other diseases [5, 8]. The BAG has also been associated with smoking, alcohol consumption [9], and measures of physical function, highlighting the value of BAG for understanding determinants of brain health.

Additionally, there is an increased interest in the use of proteomics and proteomic clocks to investigate human aging. Protein concentrations, measured with aptamer-based, SOMAscan, or mass spectrometry platforms, are the key signaling molecules in health and disease and lie more proximal to biological processes and organ function than does methylation. Proteomic clocks have been developed by several groups [10–13] which are able to accurately predict human chronologic age [10]. Other groups have reported proteomic signatures predictive of different phenotypes such as frailty [14–17], mobility disability [18], and incident dementia [19, 20]. However, fewer research groups have linked large-scale proteomic data to brain structural MRI. Shi and colleagues identified plasma proteins related to hippocampal volume and correlated with white matter hyperintensities in cognitively normal individuals [21]. Based on data from the Atherosclerosis Risk in Communities (ARIC) study, Walker and colleagues reported 38 proteins to be associated with incident dementia [19]. Harris and colleagues found that, among 90 neurology-related proteins in the Lothian Birth Cohort, the associations between tumor necrosis factor receptor superfamily member 27 (EDA2R) and general fluid cognitive ability were mediated by total brain volumes [22]. We recently investigated associations between an MRI measure of dementia risk called the AD Pattern Similarity (AD-PS) score [17–19], with 32 proteins available in the ARIC study [23] previously linked to aging in multiple studies [10]. We found the growth differentiating factor-15 (GDF-15) and pleiotrophin (PTN) proteins to be associated with the AD-PS dementia risk scores in cognitively normal individuals. However, links between large-scale proteomics and MRI measures of brain age estimation have been to our best knowledge less or not explored. In this work, we introduce a machine learning approach based on high-dimensional elastic net regression [24] to generate the BAG in ARIC participants with available MRI scans at visit 5 of the study. Once generated, to validate our method, we investigated its associations with mortality, cognitive status, physical function, risk factors, and diseases. Finally, we link this measure of brain age with proteomic data.

## Methods

Two datasets were utilized for this study. ARIC is the main target cohort, and Alzheimer’s Disease Neuroimaging Initiative (ADNI) MRI data was used to train machine learning algorithms to derive MRI-based BAG values from ARIC participants.

The ADNI was launched in 2003 as a public–private partnership. The primary goal of ADNI has been to test whether serial MRI, PET, other biological markers, and clinical and neuropsychological assessments can be combined to measure the progression of mild cognitive impairment (MCI) and early AD. The ADNI study provides a rich and well characterized cohort of cognitively normal participants and AD patients, which we used previously [25–27]. The ADNI data are described in the supplemental materials.

The ARIC study conducted the baseline exam from 1987 through 1989 among 15,792 participants mostly White and African American aged 45–64 years who were recruited from four field centers located in Forsyth County, NC; Jackson, MS; Minneapolis suburbs, MN; and Washington County, MD. Using probability sampling, each ARIC field center recruited approximately 4000 individuals from their community. Only African Americans were recruited in Jackson, MS; participants in the remaining sites were mostly White in Minneapolis and Washington County and both races in Forsyth County. The participants have completed 9 visits. Due to small sample sizes, we excluded African American participants from the Minneapolis and Washington County centers (*N* = 8). The Institutional Review Boards from all centers approved ARIC protocols; participants provided written consent. Our analysis included cohort data available through ARIC visit 5 (occurring between 2011 and 2013) who additionally have MRI and cognitive data available (*N* = 1849).

### ARIC data

#### Cognitive evaluation

The cognitive status (nonimpaired, MCI, or dementia) of participants who attended visit 5 was determined using a standardized algorithm based on a comprehensive in-person cognitive assessment, the Clinical Dementia Rating scale, and Functional Activities Questionnaires completed by participants and/or informants. Algorithmic diagnosis was verified by expert committee review [28].

### MRI

The ARIC visit 5 (2011–2013) brain MRI scans were performed on four 3 T scanners (Maryland: Siemens Verio; North Carolina: Siemens Skyra; Minnesota: Siemens Trio; Mississippi: Siemens Skyra). The following sequences were obtained: localizer, magnetization-prepared rapid gradient-echo MPRAGE (1.2-mm slices), axial gradient recalled echo T2-weighted imaging (T2*GRE) (4-mm slices), axial T2 fluid-attenuated inversion recovery (FLAIR) (5-mm slices), field mapping (3-mm slices), and axial diffusion tenor images (2.7-mm slices for Skyra and Verio scanners and 3-mm slices for Trio scanner). T2 FLAIR and T2*GRE sequences were also collected to assess brain lesion burden. The generation of the BAG values was based on the T1-weighted MPRAGE images. Image processing details have been reported previously [29, 30]. A brief description can be found in the supplementary materials.

### Mortality information

Ascertainment of mortality was based on medical records and National Death Index searches. Due to lack of access to records at one large Jackson Hospital in 2018 and 2019, we excluded from the final datasets any hospitalizations for Jackson participants for 2018 and 2019; thus, the value for the administrative censoring was set to be December 31, 2017, for Jackson participants, instead of December 31, 2019, for participants from the other three field centers.

### Health and disease measures

Physical function measures were collected as part of Short Physical Performance Battery (SPPB). In the gait speed test, participants walked 4 m at their usual pace twice, with the time of the faster trial recorded. Grip strength (kilograms) was assessed in participant’s preferred hand using an adjustable, hydraulic grip strength dynamometer with the better of two trials used in the analysis. We used the binary variable low grip strength based on the bottom gender- and body mass index–specific grip strength quintile used previously as a component of a frailty index in ARIC [31]. Prevalent stroke, heart failure, atrial fibrillation, and diabetes at the end of visit 5 were available in the ARIC database.

### Protein measurements

Full details about proteomic data collection and processing have been previously reported [19]. Briefly, using blood collected at ARIC visit 5, the relative concentration of plasma proteins or protein complexes was measured using a SOMA aptamer–based capture array. This method uses short single strands of DNA with chemically modified nucleotides, called modified aptamers, which act as protein-binding reagents with defined three-dimensional structures and unique nucleotide sequences that are identifiable and quantifiable using DNA detection technology. The SOMAscan assay has been described in detail previously, as have the assay’s performance characteristics. Plasma was collected using a standardized protocol at each ARIC site, frozen at – 80 °C, and shipped on dry ice to the ARIC central laboratory where it was continuously frozen until aliquoting into barcoded microtiter plates with screw-top lids. The plates were sent to SomaLogic for quantification. In total, 5284 modified aptamers (SOMAmers reagents or “SOMAmers”) were used to measure relative protein concentration. From those 5284 proteins, 4877 passed ARIC quality control.

### Estimation of the brain age gap

The overall approach to estimate the ARIC BAG scores is conceptually similar to the one we used before to estimate the AD-PS score [32, 33], which has been previously described [23, 29, 30]. The approach is a combination of MRI image processing methods available in the Advanced Normalization Tools (ANTs) software and the fitting of high-dimensional (voxel-based) models using the glmnet library in R library [34, 35]. The main difference with respect to previous work is that we are dealing with a high-dimensional regression problem where age is the outcome of the model. The basic steps were (1) warping the MRI images from both studies (ADNI and ARIC) into a common template (derived from ADNI images [30]) and generating the corresponding gray matter (GM) probability maps using ANTs software package [29, 30]; (2) the GM maps corresponding to 584 CN ADNI participants (Table S1) were vectorized and stacked into a matrix; (3) the matrix and age values were provided to the glmnet R library to train the elastic net regression model using chronological age as outcome; (4) GM probability maps from ARIC participants were provided as input to the elastic net model to generate an estimated age for each individual; and (5) the BAG values were estimated as the difference between estimated age and chronological age.

To estimate the model, we fixed the hyperparameter α to be 0.5. To select the value of λ, we combined tenfold cross-validation and grid search. We selected the maximum value of λ such that error is within one standard error of the minimum cross-validated error [36]. As measures of performance, the mean absolute error (MAE), the root mean squared error (RMSE), and Pearson correlation were used. To account for variability due to random partitioning of cross-validation that occurred during model estimation, these procedures were repeated 5 times and the average value of λ was used to fit the final model. To deal with known bias effects in models estimating age [37, 38] (e.g., regression dilution, non-Gaussianity of the age distribution, and regression towards the mean), we used a linear bias correction approach [39].

### Analyses

Linear regression methods were used to investigate BAG differences between cognitive groups. To investigate associations of the BAG values with mortality, Cox proportional hazards regression was performed. Participants were stratified by tertiles according to BAG values. The highest tertile with larger BAG values was treated as reference. Additional sensitivity analyses were performed using only the CN individuals and treating causes of death directly related to the central nervous system (CNS) (e.g., strokes and dementia) and non-CNS causes of death as competing risks [40]. We also investigated in separate linear regression models the associations between BAG (outcome) and diabetes, BMI, prevalent cardiovascular disease, and measures of physical function, adjusting by age, sex, and center-race.

We evaluated associations of the BAG values with 4877 SOMAscan proteins in cross-sectional analyses. We fitted linear regression models for each protein at a time using the BAG values as the outcome. A Bonferroni correction (α < 0.05, corrected) for multiple comparisons was applied. All analyses were adjusted for age, sex, center-race, smoking, hypertension, education, diabetes, and intra-cranial volume. The 5-level center-race variable (Forsyth-AA, Forsyth-W, Jackson-AA, Minn-W, Wash Co-W) was created to accommodate a lack of representation of both races in all centers.

## Results

Table 1 describes the basic demographic characteristics of the ARIC cohort who had BAG values available at visit 5 of the study (*N* = 1849), stratified by cognitive status at the time of the visit. In comparison with participants classified as having dementia or MCI, the cognitively normal group had lower prevalence of hypertension. The age range was between 67 and 90 years old.

**Table 1:** Characteristics of Atherosclerosis Risk in Communities (ARIC) study analytic sample by visit 5 cognitive status. Age and body mass index (BMI, kilograms/meter squared) mean and standard deviation values are provided. For categorical variables, sample size and percentages are presented

	Total	Cognitive status at visit 5		
		Normal	MCI	Dementia
*N*	1849	1172	589	88
Gender				
Female	1119 (60.5%)	747 (63.7%)	320 (54.3%)	52 (59.1%)
Race				
Black	538 (29.1%)	378 (32.3%)	130 (22.1%)	30 (34.1%)
Education*				
Basic	265 (14.4%)	156 (13.3%)	82 (13.9%)	27 (30.7%)
Intermediate	752 (40.7%)	449 (38.4%)	270 (45.8%)	33 (37.5%)
Advanced	830 (44.9%)	565 (48.3%)	237 (40.2%)	28 (31.8%)
Smoking status				
Current	97 (5.3%)	55 (4.8%)	37 (6.4%)	5 (6.0%)
Former	867 (47.7%)	565 (48.8%)	266 (46.3%)	36 (43.4%)
Never	764 (42.1%)	488 (42.1%)	241 (41.9%)	35 (42.2%)
Hypertension				
No	449 (24.6%)	301 (25.8%)	131 (22.6%)	17 (20.5%)
Age	76.4 (5.3)	76.0 (5.3)	76.7 (5.2)	79.3 (5.4)
Obesity (yes)	32.4%	32.6%	32.4%	32.2%
BMI	28.5 (5.7)	28.5 (5.7)	28.6 (5.7)	27.7 (5.8)

^*^Education categories: basic is less than completed high school, intermediate is high school or equivalent, and advanced is at least some college

**Fig. 1:**
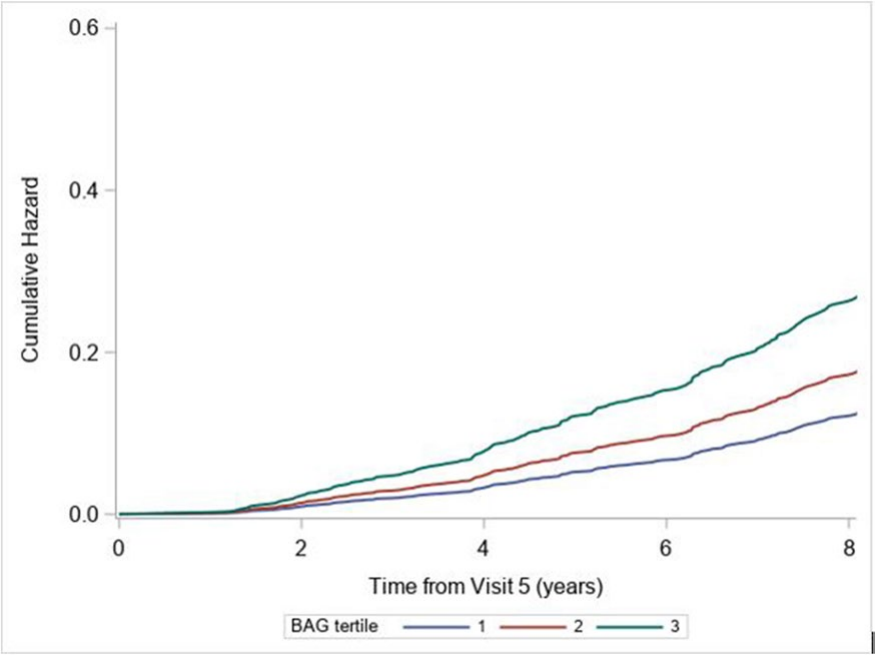
Cumulative hazards of death by tertile (*t*1 ≤  − 0.75 years, − 0.75 < *t*2 < 1.75, *t*3 ≥ 1.75) of the BAG values for cognitively normal participants at visit 5 of the study. The Cox regression model was adjusted for age, sex, center-race, smoking, hypertension, education, diabetes, and intra-cranial volume

The model’s accuracy was MAE = 2.35 years and RMSE = 3.0 while the Pearson correlation between estimated age and chronological age was 0.88. The BAG values were stratified by tertiles (*t*1 ≤  − 0.75 years, − 0.75 < *t*2 < 1.75, *t*3 ≥ 1.75). The survival analyses based on all participants reflected very strong associations of the BAG values with mortality. These associations remained significant when only CN participants were included in the analyses (see Fig. 1) and also when CNS-related and non-CNS-related causes of death were treated as competing risks (see Table 2).

**Table 2:** Associations between brain age gap (BAG) scores and all-cause mortality rates using Cox proportional hazards models adjusted for age, center-race, sex, smoking, hypertension, education, diabetes status, and intra-cranial volume. The highest tertile with larger BAG values was treated as reference

BAG score	Hazard ratio	95% confidence limits	*p*-value
All participants (*N* = 1771, deaths = 329)			
Lowest tertile (best)	0.39	(0.29–0.53)	< .0001
Middle tertile	0.60	(0.47–0.77)	< .0001
Highest tertile	1.00	-	-
Restricting to only cognitively normal participants (*N* = 1137, deaths = 163)			
Lowest tertile (best)	0.41	(0.27–0.61)	< .0001
Middle tertile	0.60	(0.42–0.87)	0.0063
Highest tertile	1.00	-	-
Restricting to cognitively normal and treating CNS-related causes of death as a competing risk (*N* = 1137, deaths = 140, competing events = 23)			
Lowest tertile (best)	0.47	(0.30–0.74)	0.0011
Middle tertile	0.66	(0.45–0.98)	0.0409
Highest tertile	1.00	-	-

**Fig. 2:**
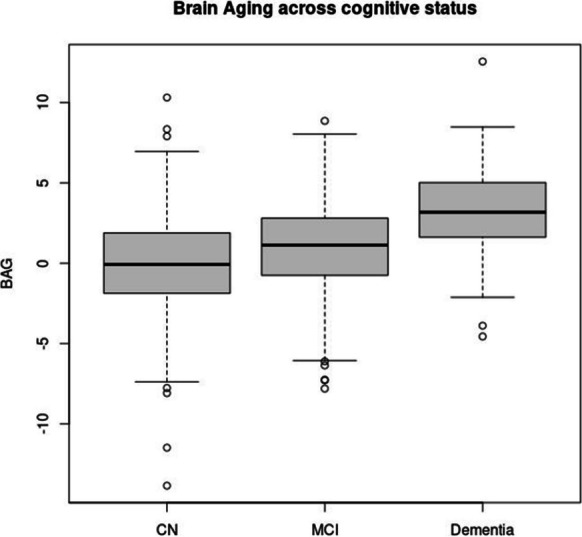
BAG across cognitive status at visit 5 of the study

We found in ARIC participants that differences in BAG values between CN-MCI and MCI-dementia participants were highly significant (Table 3). Participants with MCI and dementia had, on average, a BAG that was 0.95 and 3.16 years greater, respectively, than that of cognitively normal individuals (see Fig. 2). Further, we investigated BAG cross-sectional associations with measures of physical function, risk factors, and disease. We found the BAG values positively associated with time to walk 4 m, lower grip strength, and BMI after adjusting by age, sex, and center-race. BAG was also positively associated with diabetes, hypertension, coronary heart disease, atrial fibrillation, stroke, and heart failure (see Table S2). These associations remained significant in CN individuals with the exceptions of BMI and heart failure (see Table 4).

**Table 3:** Brain age gap (BAG) least square mean values across cognitive status and their estimated difference are presented

BAG by cognitive status groups		*N*	LSMEAN	stderr	95% CI
BAG^DEM^		88	3.27	0.29	(2.71–3.84)
BAG^MCI^		589	1.06	0.12	(0.82–1.30)
BAG^CN^		1172	0.11	0.09	(− 0.07–0.29)
Comparing cognitive status groups	*N*_1_	*N*_2_	Est. difference	stderr	*p*-value
BAG^CN^ vs. BAG^MCI^	1172	589	0.95	0.14	< .0001
BAG^DEM^ vs. BAG^MCI^	88	589	2.21	0.31	< .0001
BAG^DEM^ vs. BAG^CN^	88	1172	3.16	0.30	< .0001

BAG^DX^ refers to the BAG values in each cognitive status group

**Table 4:** Associations of the brain age gap (BAG) values with measures of physical function and disease for cognitively normal individuals only using linear regression adjusted for age, sex, and center-race

Parameter	Coefficient	95% CI limits	*p*-values
BMI	0.02	(− 0.01–0.05)	0.11
Diabetes	0.89	(0.54–1.25)	< .0001
Hypertension	0.89	(0.51–1.26)	< .0001
Time to walk 4 m	0.28	(0.27–0.52)	< .0001
Low grip strength	0.62	(0.21–1.03)	0.003
Heart failure	0.51	(− 0.07–1.09	0.12
Atrial fibrillation	1.05	(0.30–1.79)	0.006
CHD	1.10	(3.60–0.50)	0.0003
Stroke	1.54	(2.99–0.53)	0.003

Low grip strength is defined as a gender- and BMI-specific grip strength in the lowest quantile of ARIC participants [31]

*BMI* body mass index, *CHD* coronary heart disease

**Fig. 3:**
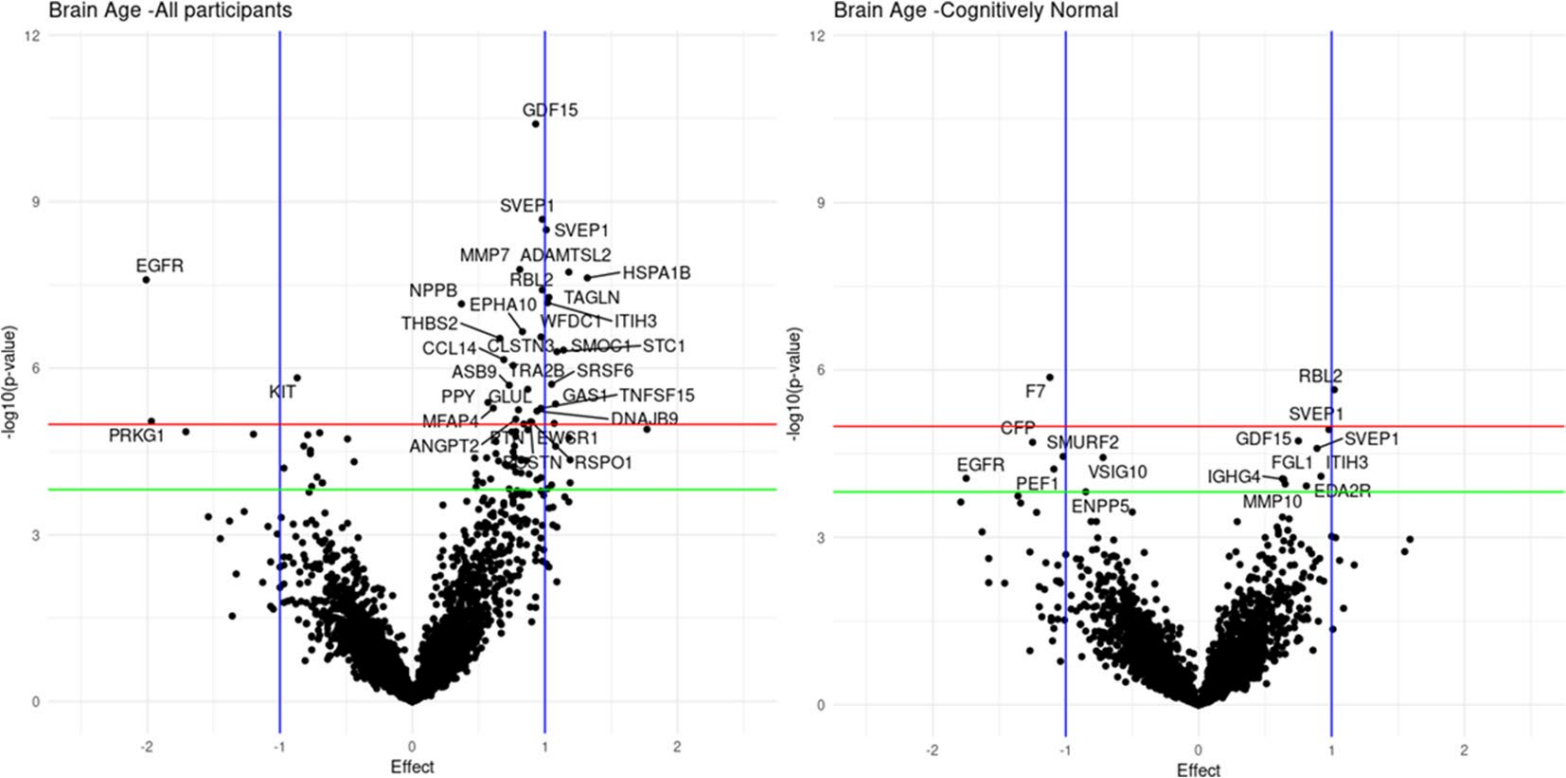
Proteomic associations with BAG of all participants (left panel) and cognitively normal (right panel) participants. We fitted linear regression models for each protein at a time using the BAG values as the outcome. A Bonferroni correction (α < 0.05, corrected) for multiple comparisons was applied. The models were adjusted for age, sex, center-race, smoking, hypertension, education, diabetes, and intra-cranial volume. Red and green horizontal lines correspond to Bonferroni and FDR correction for multiple comparisons, respectively

Finally, we investigated associations of proteomics with the BAG measure. In total, 1507 ARIC participants had both MRI and SOMAscan proteomics: 938 were CN, 495 had MCI, and 74 had dementia. Of the 4877 plasma proteins measured, we found that 33 proteins were significantly associated with BAG after the correction for multiple comparisons (see Fig. 3 and Table S3 in Supplementary material for the full list). Among these Sushi, von Willebrand factor type A, EGF, and pentraxin domain-containing protein 1 (SEVP1), growth/differentiation factor 15 (GDF-15), matrilysin (MMP7), natriuretic peptides, and heat shock 70 kDa protein 1B (HSPA1B) were found to be positively associated with the BAG, whereas EGF-receptor (EGFR), mast/stem-cell-growth-factor-receptor (KIT), and cGMP-dependent-protein-kinase-1 (PRKG1) were negatively associated with the BAG. The analysis, based only on CN participants, produced two significant proteins after the correction for multiple comparisons: retinoblastoma-2 (RBL2), which was positively associated with the BAG and coagulation-factor-VII (F7), which was negatively associated with the BAG (Table S4).

**Fig. 4:**
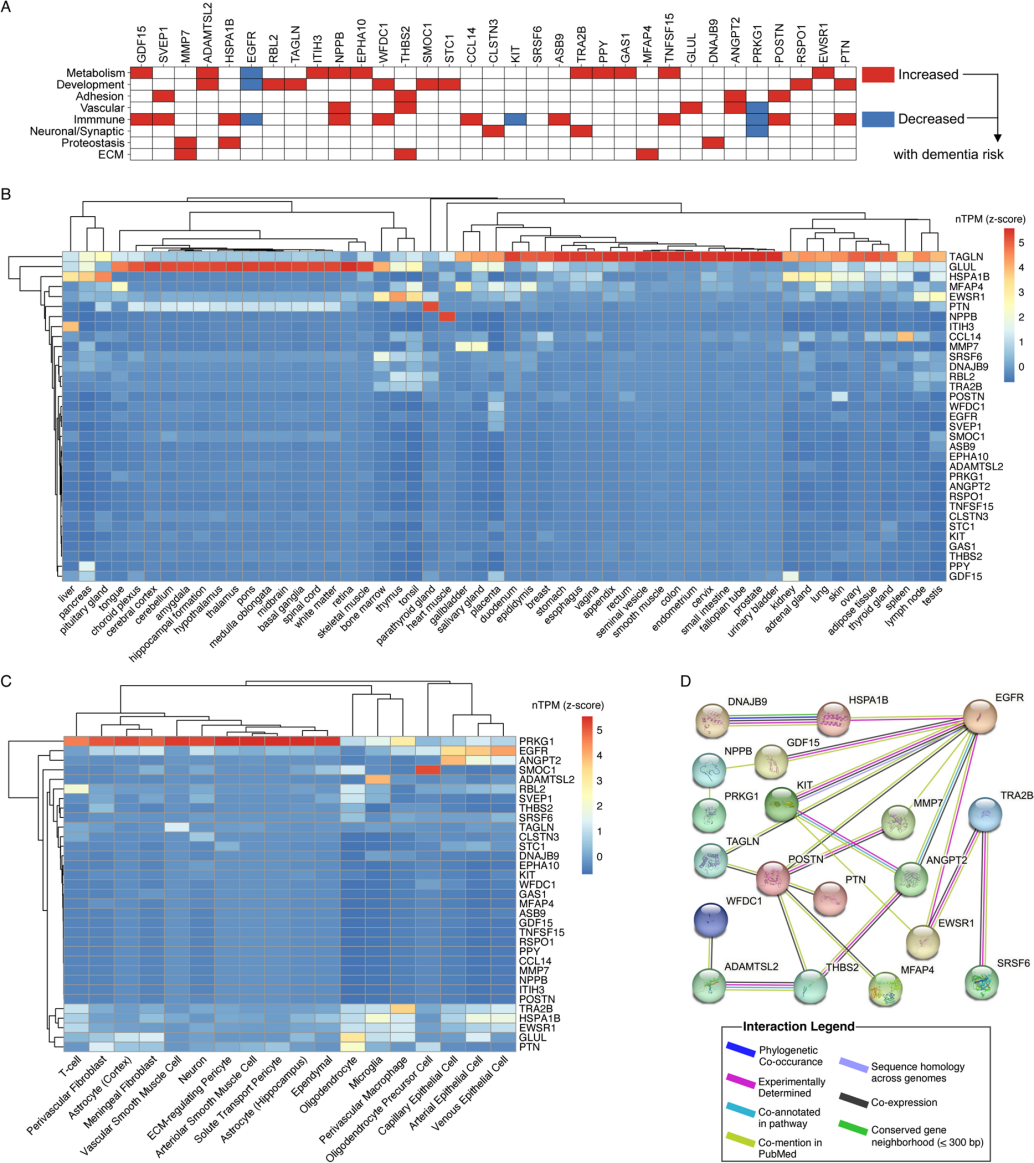
Biology of individual proteins. **A** The majority of BAG-associated proteins were implicated in one of eight biological pathways as identified by Gene Ontology (GO) terms. **B** Heatmap shows expression levels of genes encoding candidate proteins (cognate genes) across 76 available tissue types based on single-cell transcriptomics data sourced from the Human Protein Atlas. **C** Heatmap shows expression levels of genes encoding candidate proteins (cognate genes) across 18 different neurovascular cell types based on single-cell transcriptomics sourced from the Human BBB. Dendrograms reflect hierarchical clustering using Euclidean distances calculated from normalized transcripts per million (nTPM). nTPMs used to generate heatmaps were additionally standardized within cell types to improve interpretability. **D** Protein–protein interaction networks generated using STRING, with predicted conformations of proteins depicted in circular nodes

Annotation of genes encoding these proteins (i.e., cognate genes) suggested that they are involved in a variety of biological processes, including metabolism (GDF15, ADAMTSL2, ITH3), tissue development (RBL2, TAGLN), and immune function (CCL14, TNFSF15) (Fig. 4A, Table S5). Several of these proteins have been implicated previously in diseases such as cancer, schizophrenia, and heart disease, as well as several other prominent health conditions (Table S6). Using tissue expression data from the Human Protein Atlas, we found that no cognate genes showed evidence for enriched or enhanced expression within the central nervous system (Fig. 4B and Table S7). Despite limited expression in the CNS, we observed varying degrees of expression of genes coding for BAG-associated proteins across neurovascular cell types [41]. While expression for approximately half of the genes coding for BAG-associated proteins (including the top protein, GDF-15) was not detectable or very low (< 0.010 averaged normalized counts) in brain or neurovascular cell types, SVEP1 and PTN were found to primarily be expressed by oligodendrocytes, while ADAMTSL2 and HSPA1B showed strongest expression in microglia (Fig. 4C, Table S8). Notably, six of 13 (46%) BAG-associated proteins and 24 of the 33 (73%) genes coding for BAG-associated proteins were found to be differentially expressed in brain tissue of individuals diagnosed with AD, compared to control brains (Table S9). Two BAG-associated proteins that were differentially expressed at both the RNA and protein level in AD brains (SMOC1 and PTN) were previously nominated as AD therapeutic targets by the Accelerated Medicine Partnership for AD. A recent report found that SMOC1 a CSF-derived and associated with Aβ plaque protein was elevated in autosomal dominant AD nearly 30 years before the onset of symptoms [42].

## Discussion

The current study used structural MRI scans to estimate brain age, from which the deviation between estimated brain age and chronological age (BAG) was computed in a bi-ethnic community–based cohort of older adults.

We examined associations of our BAG measure with mortality risk, cognition status, prevalent diabetes and cardiovascular disease, BMI, and measures of physical function. We found the BAG values to be strongly associated with mortality risk over 8 years among ARIC participants. The associations with mortality persisted in cognitively normal individuals even when CNS-related and non-CNS-related causes of death were treated as competing risk after adjusting for several covariates reported before in a predominantly. Associations with mortality of a BAG measure have been reported before in a predominantly White cohort [8]. Since the majority of causes of death were not brain related, this suggests, as noted by Cole and colleagues, that BAG could be capturing systematic effects of aging in other organs in the human body [5]. Consistent with several previous studies, we found strong associations of BAG associations with cognitive status [1, 43].

The present study also examined the associations of BAG with measures of physical function, cardiovascular and cardiometabolic disease, and other age-related physiological variables. We found increased BAG values to be associated with increased BMI, diabetes, and hypertension prevalence and decreased grip strength and gait speed. Both gait speed and grip strength have been found to be associated with BAG in ~ 73-year-old participants of the Lothian Birth Cohort 1936 [5, 8, 44]. BMI has been found a risk factor for accelerated brain aging in first episode psychosis patients [45, 46], and diabetes has been linked to brain accelerated aging by several reports [47–50]. Franke et al. found hypertension not to be associated to BAG [49], but Beck et al. found that systolic blood pressure was associated with the BAG values [51]. They also reported associations between smoking, pulse rate, C‐reactive protein, and BAG. Associations of blood pressure were also reported using data of the UK BIOBANK but not BMI [50]. While there are multiple publications linking cardiovascular health and disease to the brain [52–54], we found only one report linking prevalent heart diseases to a BAG-like measure. Rauseo et al. very recently reported that prevalent ischemic heart disease (IHD) and vascular risk factors are associated with accelerated brain aging estimated based on a Bayesian ridge regression model built using UK Biobank participants with no prevalent IHD (*n* = 35,237) [55]. Findings from the present study provide additional support to this association between heart diseases and accelerated brain age. We found the BAG to be associated with prevalent atrial fibrillation, CHD, and stroke.

In addition to demonstrating that increased BAG is associated with several adverse age-related outcomes, we identified a circulating proteomic signature associated with BAG. These proteins included dementia-associated proteins. For example, Walker et al. reported that several proteins identified here as BAG-associated were also associated with risk for incident dementia across multiple cohorts, including the ARIC study [19]. Notably, in a recent analysis, the top BAG-associated protein, GDF15, a cytokine involved in macrophage inhibition via TGF-ß signaling, also showed the strongest association with 25-year dementia risk when measured in blood of middle-aged adults [56]. Additionally, SVEP1—an immunologically relevant cellular adhesion protein—has been causally linked to Alzheimer’s disease and multiple forms of cardiovascular disease and has been prominently associated with increased age [19, 57, 58]. Proteins associated with resilient brain aging included EGFR, a growth factor receptor that participates in Signal Transducer and Activator of Transcription (STAT) transcription factor signaling, and hemostasis-related protein coagulation factor VII (F7). EGFR has been shown to play a role in regeneration and maintenance of the CNS and also in the onset neurodegenerative diseases [59]. Recently, it has been inversely associated with dementia risk at midlife [56], and the *EGFR* gene has been implicated in Alzheimer’s disease [60]. Plasma levels of prothrombotic clotting factors like F7 are thought to increase with advancing age and with thrombotic disorders. However, downregulation of the coagulation pathway and lower levels of another coagulation factor (F10) have been associated with dementia risk among older adults [19].

Together, these results suggest that proteins involved in inflammation and cell senescence secretome (GDF15, SVEP1, EGFR, MMP7, RBL2), coagulation (F7), proteostasis (DNAJB9, HSPA1B), and angiogenesis (ADAMTSL2) are associated with accelerated brain aging.

Although none of the BAG-associated proteins was uniquely expressed within the CNS, many of them demonstrated at least some expression within brain tissue or brain cell types, including oligodendrocytes (e.g., SVEP1 and PTN) and microglia (e.g., ADAMTSL2 and HSPA1B). We therefore suspect that a subset of the BAG-associated proteins provides a readout of neurobiological processes relevant to brain aging. Importantly, proteins need not be expressed within the brain to be affected by, or to exert an effect, on brain age. Certain proteins in peripheral circulation have been shown by way of heterochronic parabiosis studies to exert pathogenic or protective effects on brain health [61, 62]. Similarly, considerable evidence suggests that proteins in blood, particularly cytokine and chemokines, can influence target cells within the brain either directly via transmigration through the blood–brain barrier, or indirectly via signaling of brain endothelial cells, or through other conduits such as the choroid plexus [63–65]. Blood–brain barrier disruption, neuroinflammation, and reduced synaptic plasticity represent just some of the neurobiological processes that can be driven by proteins outside the CNS [61, 64, 65]. However, it remains to be seen whether augmentation of BAG-associated proteins—individually or in aggregate—represents a viable therapeutic strategy for counteracting brain aging.

Model estimation in our case was not stratified by sex. We proceeded as other groups have done in the past using one model estimated based on data from both males and females [66–69]. However, recently, several groups have estimated sex-specific models [66–69]. For example, Sandford and colleagues have found using this stratified approach that associations of the brain age gap with several phenotypes were often different across sex suggesting improvements in interpretability and accuracy of results [70]. We did not have sufficient sample size to train independent models.

Strengths of this study are the use of a large and ethnically diverse cohort with decades of follow-up such as ARIC to estimate brain age and investigate its associations with multiple aging, disease, and health parameters. Most previous studies assessing the value of this type of metrics were based on predominantly White cohorts. To our best knowledge, no studies have linked large-scale proteomics to a measure of brain age such as BAG. Ours is probably among the first to have used this type of brain imaging phenotype to investigate large-scale proteomic-brain relationships. Our study is not without limitations. While ARIC is an ethnically diverse cohort, the sample from ADNI we used to train our machine learning models was not (94% White). The impact of this choice warrants future research investigating the selection of training samples. However, our results here and in our previous work on measures of AD risk in ARIC are encouraging [23, 30]. On the other hand, the range of ages of individuals whose MRIs were used for training of the machine learning algorithms was narrower with respect to other published models [43, 71] which it is unlikely to produce accurate estimates of brain age in young individuals. We were not able to determine the brain regions driving the prediction. The maps produced by the model were sparse and difficult to interpret which will require further investigation. Most of our analyses were cross-sectional. Finally, the administrative censoring for mortality was different in one of the sites mostly composed of African American participants (2017 in Jackson versus 2019 in the rest of the sites).

Different approaches have been proposed to estimate brain age, including relevance vector regression [1], support vector regression [72], Gaussian process regression (GPR) [73], and more recently convolutional neural networks (CNNs) [43, 71, 74, 75]. Here, to estimate the BAG, we used a voxel-based approach based on a high-dimensional elastic net regularized linear regression model, which is a variation of our methodology previously developed to estimate measures of dementia risk [29, 30]. Recently, elastic net regression has been compared to machine learning methods [76, 77] to estimate brain age, and, in general, it performs well with respect to nonlinear methods. However, these versions of the elastic net were based on parcellations of the brain MRI images while ours is voxel-based. These reports focused on the evaluation of the accuracy of multiple machine learning algorithms when estimating age without further investigating associations with health measures.

## Conclusions

We have estimated brain age using machine learning to determine the gap between chronologic age and brain age in ARIC, an ethnically diverse cohort finding strong associations with different aging, health parameters, and proteomics. We found strong associations of our brain age estimates with mortality, cognitive status, physical function diabetes, and prevalent heart disease. Additionally, we identified a group of proteins associated to our BAG measure in a large-scale analysis including 4788 proteins derived by a SOMA platform. Further, several of these proteins were previously associated with incident dementia in ARIC.

### Supplementary Information

Below is the link to the electronic supplementary material.

Supplementary file1 (DOCX 41 KB)
