# Supplementary material for: Associations of plasma proteomics and age-related outcomes with brain age in a diverse cohort
Source: GeroScience. 2024 Mar 4;46(4):3861–73. doi: 10.1007/s11357-024-01112-4 (PMC11226584; doi:10.1007/s11357-024-01112-4)
Supplement: Supplementary file 1 — Supplementary file1 (DOCX 41 KB) [file 11357_2024_1112_MOESM1_ESM.docx]

**ADNI database**

The Alzheimer’s Disease Neuroimaging Initiative (ADNI) was launched in 2003. Its primary goal was to test whether serial MRI, PET, other biological markers, and clinical and neuropsychological assessment could be combined to measure the progression of MCI and early AD. From over 50 sites across the U.S. and Canada, in its first phase ADNI (ADNI-1) recruited 819 participants, aged 55 to 90 years, including 229 cognitively normal (CN) individuals to be followed for 3 years, 398 people with MCI to be followed for 3 years, and 192 people with early AD to be followed for 2 years.

**MRI Data:** In this work, we used ADNI structural MRI data from 584 cognitively normal participants (See Table S1) from ADNI collected during the first visit. MRI scans were collected using 3D MPRAGE sequences, as described in the ADNI acquisition protocol [1, 2]. The ADNI protocol acquires 2 repeated scans of structural MRI data at each visit that are rated for image quality and artifacts by ADNI investigators. To enhance standardization across sites and platforms, the best dataset undergoes additional pre-processing, including corrections for gradient non-linearity and intensity non-uniformity. The ADNI study provides an excellent clinical characterization of the participants’ cognitive status and has been widely used by the neuroimaging community in multiple applications including machine learning. In this work we used MRI images from cognitively normal individuals and AD patients to train the machine learning algorithms.

**Protein characterization**

For comparative gene expression across 54 tissues we used consensus transcript expression levels (normalized Transcripts per Million; nTPM) from the Human Protein Atlas (HPA; <https://www.proteinatlas.org/about/>). We used the HPA to determine whether cognate genes for BAG-associated proteins were tissue enriched and tissue enhanced. A gene is considered *tissue enriched* if the normalized transcripts per million (nTPM) in a particular tissue type is at least four times that of any other tissue type. A gene/protein is considered *tissue enhanced* if the nTPM of the gene in 1-5 tissues is expressed at least four times the mean of other tissues[3]. Expression levels in neurovasculature cell types were obtained from the Human BBB, a transcriptomic dataset generated using VINE (Vessel Isolation and Nuclei Extraction)-seq[4] (<https://twcstanford.shinyapps.io/human_bbb/>). Protein interaction networks were assessed using STRING (Search Tool for the Retrieval of Interacting Genes/Proteins), a tool that integrates publicly available sources of information to provide a comprehensive understanding of protein–protein interaction (PPI) networks (<https://string-db.org>). Functional annotations for individual proteins obtained from STRING were powered by publicly available, open-source databases. The top three disease associations for individual proteins were reported from MalaCards, an integrated database of human maladies and their annotations (<https://www.malacards.org>). Information relevant to AD (brain tissue expression levels (RNA, protein), nominated therapeutic targets) was obtained from the Accelerating Medicines Partnership Program for AD (AMP-AD) via the AD Knowledge Portal (<https://adknowledgeportal.synapse.org>). As no separate gene IDs were available for both SomaScan SVEP1 aptamers, cognate gene identification used one gene ID (SVEP1).

Table S1 – Characteristics of ADNI participants.

|  | Cog. Normal (N = 584) |
| --- | --- |
| Age | 75.1 (55.1 – 90.4) |
| Sex (Female) | 149 (49.2%) |
| Education | 16.2 |
| Race Category | White (94%) |
| APOE | 89 (29.6%) |
| MMSE | 29.1 |
| FAQ | 0.1 |

Table S2 – BAG values associations with measures of physical function and disease for all participants.

| Parameter | Estimate | SD | 95% CI | | p-value |
| --- | --- | --- | --- | --- | --- |
| BMI | 0.03 | 0.01 | 0.01 | 0.06 | 0.004 |
| Diabetes | 0.85 | 0.14 | 0.57 | 1.13 | <0.001 |
| Hypertension | 0.91 | 0.16 | 0.60 | 1.22 | <0.001 |
| Time to walk 4m | 0.32 | 0.04 | 0.27 | 0.40 | <0.001 |
| low grip strength | 0.88 | 0.16 | 0.57 | 1.19 | <0.001 |
| Heart failure | 0.67 | 0.23 | 0.23 | 1.11 | 0.003 |
| Atrial fibrillation | 1.17 | 0.28 | 0.63 | 1.71 | <0.001 |
| CHD | 0.84 | 0.23 | 0.39 | 1.30 | <0.001 |
| Stroke | 1.20 | 0.42 | 0.38 | 2.02 | 0.004 |

Table S3 – List of proteins significantly associated with BAG in all participants after Bonferroni correction for multiple comparisons.

| **Proteins** | **EstCoef** | **EstErr** | **Tval** | **Pval** | **Annot.** |  |
| --- | --- | --- | --- | --- | --- | --- |
| Growth/differentiation factor 15 | 0.93 | 0.14 | 6.65 | 3.99E-11 | GDF15 |  |
| Sushi, von Willebrand factor type A, EGF and  pentraxin domain-containing protein 1 | 0.98 | 0.16 | 6.03 | 2.08E-09 | SVEP1 |  |
| Sushi, von Willebrand factor type A, EGF and  pentraxin domain-containing protein 1 | 1.01 | 0.17 | 5.96 | 3.21E-09 | SVEP1 |  |
| Matrilysin | 0.81 | 0.14 | 5.68 | 1.67E-08 | MMP7 |  |
| ADAMTS-like protein 2 | 1.18 | 0.21 | 5.66 | 1.86E-08 | ADAMTSL2 |  |
| Heat shock 70 kDa protein 1B | 1.32 | 0.23 | 5.61 | 2.36E-08 | HSPA1B |  |
| Epidermal growth factor receptor | -2.01 | 0.36 | -5.6 | 2.55E-08 | EGFR |  |
| Retinoblastoma-like protein 2 | 0.98 | 0.18 | 5.53 | 3.88E-08 | RBL2 |  |
| Transgelin | 1.03 | 0.19 | 5.47 | 5.29E-08 | TAGLN |  |
| Inter-alpha-trypsin inhibitor heavy chain H3 | 1.02 | 0.19 | 5.43 | 6.63E-08 | ITIH3 |  |
| Natriuretic peptides B | 0.37 | 0.07 | 5.42 | 6.95E-08 | NPPB |  |
| Ephrin type-A receptor 10 | 0.83 | 0.16 | 5.21 | 2.19E-07 | EPHA10 |  |
| WAP four-disulfide core domain protein 1 | 0.97 | 0.19 | 5.17 | 2.73E-07 | WFDC1 |  |
| Thrombospondin-2 | 0.66 | 0.13 | 5.15 | 2.89E-07 | THBS2 |  |
| SPARC-related modular calcium-binding protein 1 | 1.14 | 0.22 | 5.06 | 4.71E-07 | SMOC1 |  |
| Stanniocalcin-1 | 1.09 | 0.22 | 5.05 | 5.03E-07 | STC1 |  |
| C-C motif chemokine 14 | 0.69 | 0.14 | 4.98 | 7.02E-07 | CCL14 |  |
| Calsyntenin-3 | 0.76 | 0.15 | 4.93 | 8.93E-07 | CLSTN3 |  |
| Mast/stem cell growth factor receptor Kit | -0.87 | 0.18 | -4.83 | 1.49E-06 | KIT |  |
| Serine/arginine-rich splicing factor 6 | 1.05 | 0.22 | 4.78 | 1.94E-06 | SRSF6 |  |
| Ankyrin repeat and SOCS box protein 9 | 0.73 | 0.15 | 4.77 | 2.02E-06 | ASB9 |  |
| Transformer-2 protein homolog beta | 0.87 | 0.18 | 4.74 | 2.39E-06 | TRA2B |  |
| Pancreatic prohormone | 0.57 | 0.12 | 4.62 | 4.12E-06 | PPY |  |
| Growth arrest-specific protein 1 | 1.08 | 0.23 | 4.61 | 4.40E-06 | GAS1 |  |
| Microfibril-associated glycoprotein 4 | 0.61 | 0.13 | 4.57 | 5.22E-06 | MFAP4 |  |
| Tumor necrosis factor ligand superfamily member 15 | 0.97 | 0.21 | 4.57 | 5.26E-06 | TNFSF15 |  |
| Glutamine synthetase | 0.8 | 0.18 | 4.56 | 5.63E-06 | GLUL |  |
| DnaJ homolog subfamily B member 9 | 0.94 | 0.21 | 4.55 | 5.91E-06 | DNAJB9 |  |
| Angiopoietin-2 | 0.78 | 0.17 | 4.47 | 8.28E-06 | ANGPT2 |  |
| cGMP-dependent protein kinase 1 | -1.97 | 0.44 | -4.46 | 9.02E-06 | PRKG1 |  |
| Periostin | 0.89 | 0.2 | 4.45 | 9.26E-06 | POSTN |  |
| R-spondin-1 | 0.9 | 0.2 | 4.45 | 9.35E-06 | RSPO1 |  |
| RNA-binding protein EWS | 1.07 | 0.24 | 4.44 | 9.83E-06 | EWSR1 |  |
| Pleiotrophin | 0.84 | 0.19 | 4.43 | 1.01E-05 | PTN |  |

Table S4 - List of proteins significantly associated with BAG in cognitively normal participants after an FDR (<0.05) correction for multiple comparisons. Only the two top proteins survive the Bonferroni correction.

| **Proteins** | **EstCoef** | **EstErr** | **Tval** | **Pval** |  |
| --- | --- | --- | --- | --- | --- |
| Coagulation factor VII | -1.12 | 0.23 | -4.86 | 1.36E-06 | F7 |
| Retinoblastoma-like protein 2 | 1.02 | 0.21 | 4.76 | 2.26E-06 | RBL2 |
| Sushi, von Willebrand factor type A, EGF and pentraxin domain-containing protein 1 | 0.98 | 0.22 | 4.41 | 1.17E-05 | SVEP1 |
| Growth/differentiation factor 15 | 0.75 | 0.17 | 4.3 | 1.87E-05 | GDF15 |
| Properdin | -1.25 | 0.29 | -4.29 | 1.98E-05 | CFP |
| Sushi, von Willebrand factor type A, EGF and pentraxin domain-containing protein 1 | 0.89 | 0.21 | 4.23 | 2.55E-05 | SVEP1 |
| E3 ubiquitin-protein ligase SMURF2 | -1.02 | 0.25 | -4.16 | 3.54E-05 | SMURF2 |
| V-set and immunoglobulin domain-containing protein 10 | -0.72 | 0.17 | -4.15 | 3.70E-05 | VSIG10 |
| Peflin | -1.09 | 0.27 | -4.03 | 5.98E-05 | PEF1 |
| Inter-alpha-trypsin inhibitor heavy chain H3 | 0.92 | 0.23 | 3.96 | 8.04E-05 | ITIH3 |
| Epidermal growth factor receptor | -1.75 | 0.45 | -3.94 | 8.75E-05 | EGFR |
| Fibrinogen-like protein 1 | 0.63 | 0.16 | 3.94 | 8.90E-05 | FGL1 |
| Immunoglobulin heavy constant gamma 4 | 0.64 | 0.16 | 3.93 | 9.13E-05 | IGHG4 |
| Stromelysin-2 | 0.65 | 0.17 | 3.88 | 0.00011 | MMP10 |
| Tumor necrosis factor receptor superfamily member 27 | 0.81 | 0.21 | 3.86 | 0.00012 | EDA2R |
| Ectonucleotide pyrophosphatase/phosphodiesterase family member 5 | -0.85 | 0.22 | -3.8 | 0.000153 | ENPP5 |

**References**

1. Jack, C.R., Jr., et al., *The Alzheimer's Disease Neuroimaging Initiative (ADNI): MRI methods.* J Magn Reson Imaging, 2008. **27**(4): p. 685-91.

2. Hua, X., et al., *Tensor-based morphometry as a neuroimaging biomarker for Alzheimer's disease: an MRI study of 676 AD, MCI, and normal subjects.* Neuroimage, 2008. **43**(3): p. 458-69.

3. Uhlen, M., et al., *Proteomics. Tissue-based map of the human proteome.* Science, 2015. **347**(6220): p. 1260419.

4. Yang, A.C., et al., *A human brain vascular atlas reveals diverse mediators of Alzheimer’s risk.* Nature, 2022. **603**(7903): p. 885-892.
